# Broadband impedance matching for lossy magnetic metamaterials in conductive media

**DOI:** 10.1038/s41598-025-11452-6

**Published:** 2025-07-30

**Authors:** Connor Jenkins, Asimina Kiourti

**Affiliations:** ElectroScience Laboratory, Department of Electrical and Computer Engineering, The Ohio State University, Columbus, OH 43212 USA

**Keywords:** Engineering, Electrical and electronic engineering

## Abstract

We present a broadband impedance matching model for traveling waves on lossy magnetic metamaterials known as magnetoinductive waveguides (MIWs) in conductive media. Thus far, broadband impedance matching has only been demonstrated in the case of a lossless MIW in free-space due to complexities introduced by losses and eddy current effects. As such, current studies in conductive environments have been limited to utilizing narrow-band matching techniques or relying on attenuation to mitigate reflections, thus limiting the system performance in terms of bandwidth and transmission loss. The proposed model overcomes these limitations by utilizing the nearest neighbor coupling and binomial approximations to generate transducer design criteria in terms of equivalent circuit parameters for broadband impedance matching. To validate the model, a transducer is designed for a 40-MHz lossy MIW submerged in an ocean water phantom. Reflection coefficient results demonstrate a 15.5% fractional bandwidth and a maximum value of − 9.0 dB in the propagation band of the MIW, indicating excellent performance. This model expands the potential design space of MIWs to include complex environments such as underwater, underground, or on the human body.

## Introduction

Metamaterials, here referring to composite materials designed to achieve certain electromagnetic characteristics^1^, have been a topic of theoretical study for decades^2–5^. In relatively recent history, their study has grown to extend beyond theory and into reality through the first realizations of negative permeability and permittivity materials^6,7^ in engineered dielectrics. Traditionally, the study of metamaterials has focused on the manipulation of electromagnetic wave interactions to achieve some desired bulk effective permittivity and/or permeability^8,9^. The additional design flexibility awarded by eliminating limitations in achievable material properties has led to advances in wireless power transfer^10^, antenna design^11^, imaging^12^, and optical cloaking^13^.

In addition to the study of electromagnetic wave interactions, there has been growing interest in the study of waves supported by the metamaterial structures themselves^14–16^. In this work, we focus specifically on magnetoinductive waveguides (MIWs), a type of magnetic metamaterial that supports traveling magnetoinductive waves^17^. MIWs are created using periodically spaced, electrically small, resonant elements that are magnetically coupled to one another^17^. The structure of MIWs are not limited solely to mono-atomic geometries, containing a single resonant element per periodic unit, but have also been analyzed with di-atomic^18–20^ and poly-atomic^21^ configurations. Besides these typical geometric scenarios, MIWs have also been explored using combined^22^, multi-turn^23^, and coaxial-based^24^ elements. There has also been considerable work in defining the transmission behavior of mono-atomic MIWs under a variety of non-idealities, such as in the presence of noise^25^, conductive environments^26^, and significant higher-order interactions^27^. Such studies have further expanded to the waves themselves, which have been well-characterized through analyses of power waves and scattering parameters^28^, wave relations^29^, and Brilloiun diagrams^30^. With this well-defined theory, several MIW-based devices have been created. Examples include power splitters^31^, phase shifters^31^, filters^31,32^, and switchable routers^33,34^. This has led to their use in applications ranging from sensing^35–38^ to wireless power transfer^39,40^ and communications^41–43^.

In particular, there is significant interest in utilizing MIWs in traditionally challenging Radio-Frequency (RF) environments, such as underground^44^, underwater^45^, or on the human body^46,47^. This is because the MIW transmission characteristics are relatively invariant to the permittivity^46^ and well-understood for the conductivity^26^ of the surrounding environment. However, the primary challenge facing mono-atomic MIW use in these environments is the inherent difficulty of impedance matching due to their complex-valued and frequency-dependent characteristic impedance^17^. Because of this complexity, broadband impedance matching has only been demonstrated for lossless, mono-atomic MIWs in free-space^48^. As the current state-of-the-art does not integrate losses, nor does it account for effects of conductive media, it has not been widely utilized^49,50^. Instead, typical approaches either focus on the transmission characteristics of the channel while relying on losses to mitigate reflections^23,24,46,47^, utilize single-frequency matching techniques^39,44^, or empirically determine optimal transducer geometry^41,42^. Expectedly, such approaches are inherently limiting the maximum performance of the MIW system in some way.

In this work, we present a broadband impedance matching model for lossy MIWs in conductive media. We overcome the limitations of existing models by robustly handling element loss, transducer loss, and the generation of eddy currents in the surrounding conductive environment via complex-valued circuit parameters. This model is summarized by three criteria that alone are sufficient to create an optimally matched MIW system. Following the derivation of these criteria, an impedance-matched MIW system operating at 40 MHz is constructed, integrated, and tested, without loss of generality, in an ocean water phantom, to validate the model. Our system achieves a − 10 dB fractional impedance bandwidth of 15.5%, with a maximum input reflection of − 9 dB in the propagation band, while demonstrating a minimum propagation loss of 1.52 dB/cm. Predicted behavior based on the equivalent circuit parameters and matching network architecture also shows excellent agreement with the measured values. These results indicate that the proposed impedance matching model and design process are highly effective at determining and predicting optimal impedance matching behavior. By utilizing the proposed model, MIW performance can be expanded considerably for near-field communication applications underwater, underground, or on the human body.

## Theoretical modeling

### Circuit model in conductive media

**Fig. 1:**
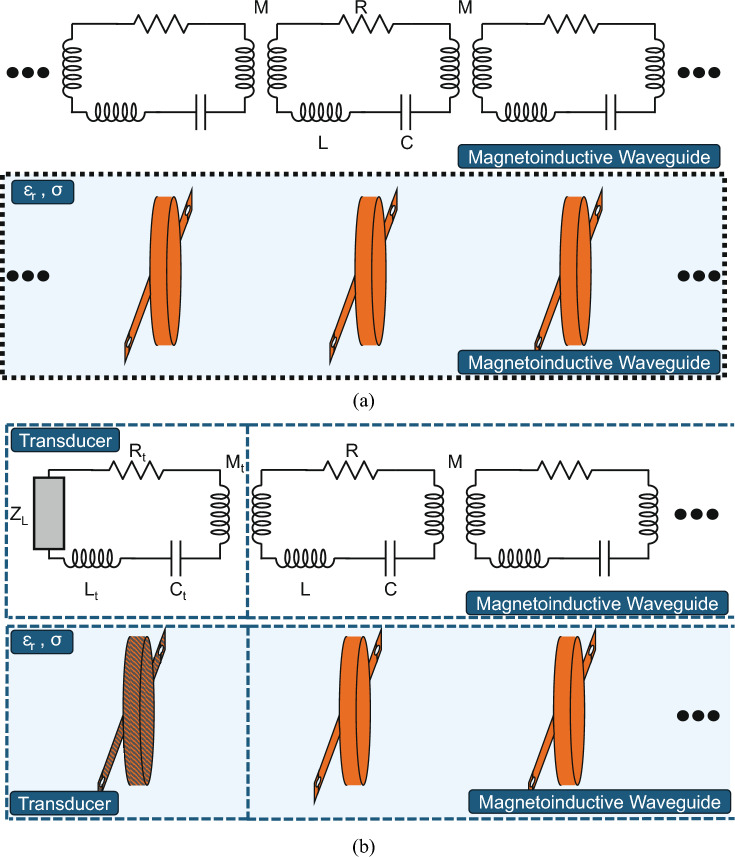
Representations of mono-atomic MIW scenarios and equivalent circuit diagrams. Each MIW element equivalent circuit consists of a resistance, *R*, capacitance, *C*, and self-inductance, *L* while being coupled to the neighboring elements via mutual inductance *M*. Each element of the MIW is pictorially represented by an encapsulated loop surrounded by a general medium with relative permittivity $\epsilon _r$ and conductivity $\sigma$ to align with the examined scenario of a lossy MIW in conductive medium. (**a**) Equivalent circuit model and pictorial representation of an infinite-length mono-atomic MIW immersed in a general medium with each element coupled solely to its nearest neighbor. Only three identical elements are shown to represent the periodicity of the structure. (**b**) Equivalent circuit model and pictorial representation of a semi-infinite length mono-atomic MIW submerged in a general medium with relative permittivity $\epsilon _r$ and conductivity $\sigma$ and terminated by a magnetically coupled transducer on one end. Two elements of the MIW are shown to demonstrate the periodicity of the structure after the termination. The transducer is not necessarily identical to the MIW elements and is represented by a resistance $R_t$, self-inductance $L_t$, and capacitance $C_t$. The transducer is loaded with an impedance $Z_L$ and magnetically coupled to the final MIW element with mutual inductance $M_t$.

To begin, we examine a lossy, uniform mono-atomic MIW with geometric period *a*, placed in a stable conductive and non-magnetic medium with finite permeability, as shown in Fig. 1a where $\epsilon _r$ and $\sigma$ are the relative permittivity and conductivity of the medium. As is typical with MIW analysis, we will assume that (1) nearest-neighbor interactions dominate, (2) the MIW is infinite in length, and 3) the elements are electrically small and made resonant at a radial frequency of $\omega _0$. In addition, we assume that the elements themselves are electrically isolated from the environment, such that no conduction current is generated in the conductive medium. With this in mind, each element is defined by an impedance of $Z = R + j\omega L + 1/j\omega C$ where *L* is the self-inductance, *C* is the capacitance, and *R* is the resistance of each element, $\omega$ is the radial frequency, and *j* is the imaginary unit. Finally, each element is coupled to its nearest neighbor by the mutual inductance *M*. To handle the eddy current effects of the surrounding conductive media, we represent the self- and mutual-inductances as complex valued Kirchhoff coefficients^26^. Specifically, we define $L = L' + jL''$ and $M = M' + jM''$, where we note that, unlike the free-space case, all values are weakly frequency-dependent. This is a slightly altered definition from previous work^26^, as we do not separate the free-space self- and mutual-inductance from the change in self- and mutual-inductance caused by the presence of eddy currents.

With the MIW assumptions and elements equivalent circuit defined, we move to the dispersion relation definition that connects the operating radial frequency $\omega$ with the complex propagation constant $\gamma$. While the dispersion relation for a mono-atomic MIW in conductive media has been previously defined^26^, we reshape the equation with our chosen definitions and additional relevant parameters as

1 $$\begin{aligned} 1 - \frac{\omega _0^2}{\omega ^2}(1+j\Lambda )^{-1} - \frac{j}{\tilde{Q}} + \kappa \cos {\gamma a}=0 \end{aligned}$$

Here, we have two algebraically convenient parameters. First, the complex-valued quality factor $\tilde{Q} = \omega L/R$, and second, $\Lambda = L''/L'$ here referred to as the cotangent factor. In addition, we have $\omega _0=1/\sqrt{L'C}$ as the resonant radial frequency of each element, $\kappa =2M/L$ as the complex-valued coupling coefficient, and $\gamma$ as the complex-valued propagation constant. We note here that Eq. (1) reduces to the free-space dispersion relation as the conductivity of the surrounding medium reduces to 0.

Next, we define the characteristic impedance of the MIW in conductive media. As is typical with transmission-line-like technology, this is defined as the terminating impedance that eliminates reflections back towards the line.

2 $$\begin{aligned} Z_0=j\omega M e^{-j\gamma a} \end{aligned}$$

This definition is seemingly identical to the free-space case, but there are two slight differences. First, the mutual inductance is complex-valued and frequency-dependent, whereas the free-space case uses a real-valued constant. Second, the propagation constant is defined by Eq. (1) rather than the free-space dispersion relation for mono-atomic MIWs^17^. Despite these changes, Eq. (2) does converge to the free-space solution as the surrounding conductivity is reduced to 0.

With the infinite-length MIW behavior defined, we then examine the case of a finite MIW with *N* elements. To terminate the MIW, we have a transducer coupled to the $N^{th}$ element with mutual inductance $M_t=M_t'+jM_t''$. The transducer is electrically small and defined by the impedance $Z_t=Z_L+R_t+j\omega L_t+1/j\omega C_t$, where $L_t=L_t'+jL_t''$, $C_t$, and $R_t$ are the self-inductance, capacitance, and resistance of the transducer, and where $Z_L=R_L+jX_L$ is the terminating load impedance placed on the transducer. The scenario is summarized in Fig. 1b. Following from previous work^48^, we can then define the effective load impedance inserted into the $N^{th}$ element in the MIW by the coupled transducer as

3 $$\begin{aligned} Z_{L,eff} = \frac{\omega ^2 M_t^2}{Z_L + R_t + j\omega L_t(1 - \frac{\omega _{0t}^2}{\omega ^2}(1+j\Lambda _t)^{-1})} \end{aligned}$$

where $\omega _{0t}=1/\sqrt{L_t'C_t}$ and $\Lambda _t=L_t''/L_t'$ are the resonant frequency and the cotangent factor of the transducer, respectively.

We now define the current reflection coefficient at the effect load as the ratio of the reflected and incident current. Following from the literature^48^, this leads to

4 $$\begin{aligned} \Gamma _{eff} = -\frac{Z_{L,eff} - Z_0}{Z_{L,eff} + Z_0^*} \end{aligned}$$

where $*$ denotes the complex conjugate. Eq. (4) makes it clear that, in order to have broadband impedance matching, we must have $Z_{L,eff}\cong Z_0$ for an appreciable bandwidth.

### Proposed impedance matching model

With the underlying MIW theory defined, our goal is to develop criteria for broadband impedance matching in terms of the equivalent circuit models of the MIW and transducer in conductive media. The derivation is similar to the lossless case^48^ with some key changes that will be highlighted throughout. First, we normalize the effective load impedance and characteristic impedance of the MIW by the approximate characteristic impedance of the MIW at resonance, $Z_0|_{\omega =\omega _0}\approx \omega _0 M$. Through careful algebraic manipulation, we have

5 $$\begin{aligned} Z_{LN,eff} = \frac{w}{\frac{\rho _T}{w\mu ^2} +j\frac{2\lambda }{\kappa \mu ^2}(1 - \frac{\eta ^2}{w^2}(1+j\Lambda _t)^{-1})} \end{aligned}$$

6 $$\begin{aligned} Z_{0N} = \frac{w}{\sqrt{1-(-\frac{1}{\kappa }+\frac{1}{\kappa w^2(1+j\Lambda )}+\frac{j}{\kappa \tilde{Q}})^2}+j(\frac{1}{\kappa }-\frac{1}{\kappa w^2(1+j\Lambda )}-\frac{j}{\kappa \tilde{Q}})} \end{aligned}$$

where $w=\omega /\omega _0$, $\mu =M_t/M$, $\lambda =L_t/L$, $\eta =\omega _{0t}/\omega _0$, and $\rho _T=(R_t+Z_L)/\omega _0M$. We then convert these normalized impedances to normalized admittances, $Y_{LN,eff}$ and $Y_{0N}$, and set them equal to one another, representing the conditions that allow for $\Gamma _{eff}=0$ per Eq. (4). This leads to the following governing broadband impedance matching equation

7 $$\begin{aligned} \sqrt{1-\left( -\frac{1}{\kappa }+\frac{1}{\kappa w^2(1+j\Lambda )}+\frac{j}{\kappa \tilde{Q}}\right) ^2}+j\left( \frac{1}{\kappa }-\frac{1}{\kappa w^2(1+j\Lambda )}-\frac{j}{\kappa \tilde{Q}}\right) -\frac{\rho _T}{w\mu ^2} -j\frac{2\lambda }{\kappa \mu ^2}\left( 1 - \frac{\eta ^2}{w^2}(1+j\Lambda _t)^{-1}\right) =0 \end{aligned}$$

In this lossless free-space case, the expression could be easily separated into real and imaginary components which facilitate the creation of matching criteria^48^. However, the inclusion of loss and the effects of eddy currents complicate the process considerably. First, because our parameters are complex-valued to account for eddy current generation in the surrounding environment, we cannot easily separate the real and imaginary parameters. Instead, the terms that include the 90°phase shift introduced by $\pm j$ will be separated from those coefficients that do not have the additional phase change. We will refer to these components as the quadrature and in-phase components, respectively. Even with this simplification, the form of our equation remains challenging - particularly the separation of the quadrature and in-phase components of the terms under the radical. To extract these terms, we utilize the binomial approximation which holds true for the following conditions

8 $$\begin{aligned} \left| \left( -\frac{1}{\kappa } + \frac{1}{\kappa w^2(1 + j\Lambda )} + \frac{j}{\kappa \tilde{Q}}\right) ^2 \right| <1 \end{aligned}$$

9 $$\begin{aligned} \frac{1}{2}\left| \left( -\frac{1}{\kappa } + \frac{1}{\kappa w^2(1 + j\Lambda )} + \frac{j}{\kappa \tilde{Q}}\right) ^2 \right| \ll 1 \end{aligned}$$

For $|\tilde{Q}|\gtrsim 100$ and operation near resonance such that $w\approx 1$ there is a proportional relationship between the minimum required $|\kappa |$ and the absolute value of the cotangent factor of the MIW element, $|\Lambda |$. Essentially, as the eddy current effects begin to dominate, the MIW requires stronger coupling in order to properly utilize this impedance matching model. Applying this approximation, we have

10 $$\begin{aligned} 1-\frac{1}{2}\left( -\frac{1}{\kappa }+\frac{1}{\kappa w^2(1+j\Lambda )}+\frac{j}{\kappa \tilde{Q}}\right) ^2 + j\left( \frac{1}{\kappa }-\frac{1}{\kappa w^2(1+j\Lambda )}-\frac{j}{\kappa \tilde{Q}}\right) -\frac{\rho _T}{w\mu ^2} -j\frac{2\lambda }{\kappa \mu ^2}\left( 1 - \frac{\eta ^2}{w^2}(1+j\Lambda _t)^{-1}\right) =0 \end{aligned}$$

By separating the quadrature and in-phase components as previously discussed, we generate the following broadband impedance matching criteria

11 $$\begin{aligned} \Lambda (1+\Lambda ^2)(1+\Lambda _t)^2=0 \end{aligned}$$

12 $$\begin{aligned} \frac{\mu ^2}{2}\left( \frac{1}{\kappa \tilde{Q}}+1\right) =\lambda \end{aligned}$$

13 $$\begin{aligned} \eta ^2=\frac{(1+\Lambda _t^2)(1/\tilde{Q} + \Lambda + \kappa )}{(1+\Lambda ^2)(1/\tilde{Q} + \kappa )} \end{aligned}$$

14 $$\begin{aligned} \begin{aligned}&w^4(1+\Lambda ^2)^2(2\kappa ^2+1/\tilde{Q}^2+2\kappa /\tilde{Q}-1) + w^3(-2\frac{\rho _T}{\mu ^2}\kappa ^2(1+\Lambda ^2)^2)+ \\&2 w^2(1+\Lambda ^2)(1+\Lambda \Lambda _t-\Lambda _t/\tilde{Q}+\Lambda /\tilde{Q} - \kappa \Lambda + \kappa \Lambda _t)+(\Lambda ^2-1)=0 \end{aligned} \end{aligned}$$

Because the cotangent factors can never be equal to 0 in the presence of conductive media, the first criterion in Eq. (11) can never be true. To improve the criteria, we instead absorb this impossible criterion into the derivation for Eq. (17) and approximate $\Lambda ^2\approx \Lambda _t^2\approx 0$ such that

15 $$\begin{aligned} \eta ^2\approx \frac{(1/\tilde{Q} + \kappa + \Lambda (1-1/w^2))}{(1/\tilde{Q} + \kappa )} \end{aligned}$$

Finally, when operating near resonance and with typical values of $|\Lambda |<1$, we have $\Lambda (1-1/w^2)\approx 0$, which leads to the final three matching criteria with $\alpha =\rho _T/\mu ^2$

16 $$\begin{aligned} \frac{\mu ^2}{2}\left( \frac{1}{\kappa \tilde{Q}}+1\right) =\lambda \end{aligned}$$

17 $$\begin{aligned} \eta ^2\approx 1 \end{aligned}$$

18 $$\begin{aligned} \begin{aligned}&w^4(1+\Lambda ^2)^2(2\kappa ^2+1/\tilde{Q}^2+2\kappa /\tilde{Q}-1) + w^3(-2\alpha \kappa ^2(1+\Lambda ^2)^2)+ \\&2 w^2(1+\Lambda ^2)(1+\Lambda \Lambda _t-\Lambda _t/\tilde{Q}+\Lambda /\tilde{Q} - \kappa \Lambda + \kappa \Lambda _t)+(\Lambda ^2-1)=0 \end{aligned} \end{aligned}$$

Note that these criteria do not fully converge to the lossless case^48^ when $\tilde{Q}\Rightarrow \infty$ and $\Lambda =\Lambda _t=0$ because of our use of the binomial approximation. However, the difference is very small when the binomial approximation criteria are met, as expected. Additionally, the approximation that $\Lambda ^2\approx \Lambda _t^2\approx 0$ is utilized when solving for Eq. (17) to greatly simplify the final criterion and illuminate the relative independence of the design to the resonant frequencies without significant loss in accuracy. Applying this same approximation to the other criterion does not greatly simplify the expressions and does not lead to better understandings of the design process and as such the other criterion are kept as exact as possible. For more detail regarding the derivation process, see Supplementary Note 1.

To examine the meaning of these criteria, we will assume that the MIW is defined while the transducer is unknown. This is not meant to limit the generality of the criteria, but rather offer some insight and understanding. First, Eq. (16) relates the geometry of the MIW to the geometry of the transducer. The self-inductance of the transducer is present in $\lambda$, while the mutual inductance between the $N^{th}$ element and the transducer is present in $\mu$. This criterion can be met by adjusting the transducer geometry and/or the location of the transducer relative to the $N^{th}$ element. Next, Eq. (17) simply states that the resonant frequencies of the elements and the transducers should be identical. Finally, Eq. (18) has three control variables in our chosen scenario: $\Lambda _t$, $\mu$, and $\rho _T$. Because $\Lambda _t$ is largely controlled by the environmental conditions and $\mu$ is an important parameter to meet the requirement in Eq. (16), $\rho _T$ is the main tuning parameter. With $\rho _T$ directly related to the load impedance on the transducer, the selection of $\rho _T$ may also be limited by additional system-level factors, particularly if the MIW is to be integrated into a traditional 50 $\Omega$ RF system.

## Results and discussion

To validate and apply this impedance matching model, we examine, without loss of generality, an underwater ocean communication scenario centered at 40 MHz and matched to a 50 $\Omega$ system. At this frequency, ocean water is defined by $\epsilon _r=74$ and $\sigma =5$ S/m^51^. We select an axially-aligned MIW design with circular elements (radius = 2 cm), such that our electrically small criteria is met (< 1/50th of a wavelength at 40 MHz). In addition, each element is separated from its neighbor by 2.25 cm, such that the second-order coupling is less than 20% of the first-order coupling, thereby achieving the nearest neighbor approximation. To simplify the problem, without loss of generality, we will also use the same 2 cm radius circular geometry for the transducer. Both the transducers and elements are encapsulated in an air cavity to eliminate conduction currents in the environment. With this MIW in mind, we first design the transducer and load impedance to satisfy Eqs. (16–18) through theoretical analysis and simulation and then analyze the full-system experimentally.

### Criteria optimization

The first step to optimizing the criteria is to extract the equivalent circuit parameters of the MIW design. Using CST Studio^52^, two encapsulated MIW elements are simulated and submerged in ocean water. At 40 MHz, this gives us $R=0.27$ $\Omega$, $L=127.95-j4.87$ nH, and $M=7.09-j2.09$ nH. Calculating the derived parameters, we have $\kappa =0.11-j0.03$, $\tilde{Q}=119.2-j4.5$ and $\Lambda =-0.04$. Note that because our transducer geometry is identical to our element geometry, we also have $R_t=R$, $L_t=L$ and $\Lambda _t=\Lambda$. In addition, while we only report the parameters at 40 MHz, the full frequency-dependent parameters are used throughout the remainder of the work. With $w=1$ and the given derived parameters, the conditions for the binomial approximation as presented in Eqs. (8–9) are met and the presented impedance matching model can be used.

We now move to the matching criteria in Eqs. (16–18). As previously discussed, each criterion can be met by 1) shifting the location of the transducer, 2) controlling the resonance of the elements and transducer, and 3) determining the optimal load impedance placed on the transducer. First, to determine the optimal location of the transducer in relation to the final element, CST Studio is used in conjunction with Eq. (16). After an iterative design process, the optimal distance that minimizes the error of Eq. (16) is found to be 1.83 cm, such that $M_t=9.92-j2.15$ nH and $\mu =1.37+j0.1$. Second, because our elements and transducers are identical, Eq. (17) is satisfied by simply loading each loop with the same capacitance, in this case 121 pF to achieve resonance at 40 MHz. Finally, with all relevant parameters defined, we can use Eq. (18) to determine the optimal load impedance for broadband impedance matching by tuning $\alpha$. Unlike the lossless case, where $\alpha$ was entirely real and shifted the roots of a real-valued polynomial^48^, we instead have a complex value of $\alpha =|\alpha |e^{\angle \alpha }$ that controls the minimum value of a complex-valued polynomial. In addition, it is not enough to select the value of $\alpha$ that minimizes Eq. (18) because the selected impedance will impact the optimal impedance matching performance of the impedance matching network connecting the MIW to a 50 $\Omega$ system as determined by the Bode-Fano Criterion. This is of particular interest in the given scenario because the load impedance will be very small, with $Z_L=\alpha \mu ^2\omega _0M=\alpha (2.58-j0.53)$ while $\alpha$ is limited to $0.7\le |\alpha |\le 1.3$ and $-\pi /12\le \angle \alpha \le \pi /12$ in order to minimize Eq. (18). With this in mind, we utilize a finite-section model of the Bode-Fano criteria^53^ in addition to Eq. (18) to determine the optimal load impedance. In particular, a 2-section matching network is selected as a tradeoff between maximum achievable bandwidth and matching network complexity while reducing the potential effects from distributed component non-idealities which are very impactful at our very low required load impedances.

**Fig. 2:**
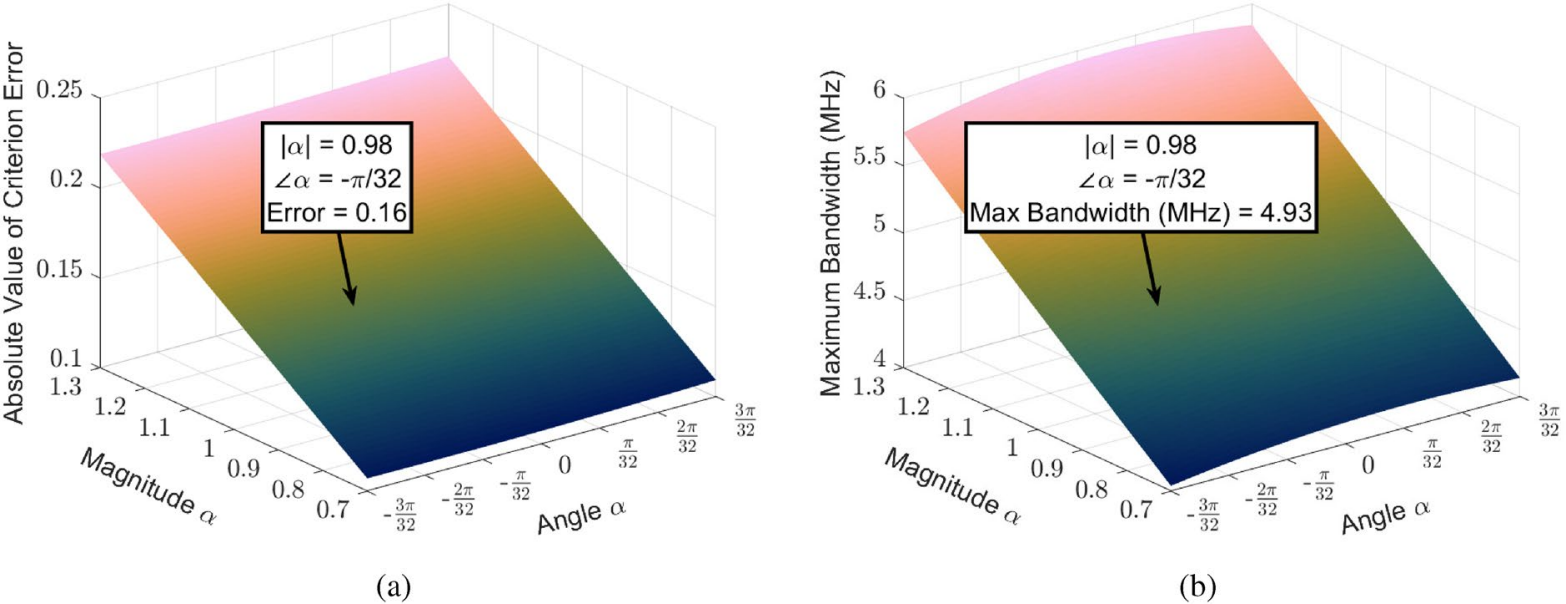
Surface plots of criteria optimization parameters versus the phase and magnitude of $\alpha$. The magnitude of $\alpha$ is limited from 0.7 to 1.3 in 64 steps while the angle is limited between $\pm 3\pi /32$ in 64 steps for a total of 4096 points. The selected magnitude and phase of $\alpha$ are labeled along with the corresponding error and maximum bandwidth metrics. (**a**) Absolute value of error in Eq. (18) for each given value of $\alpha$. (**b**) Maximum achievable bandwidth according to the adjusted Bode-Fano Criterion for a 2-section matching network with a minimum reflection coefficient of 0.1.

Figure 2 shows both the value of Eq. (18) at $w=1$ and the optimal bandwidth for a 2-section matching network with a minimum reflection coefficient of 0.1 for each given $\alpha$. It is clear that, to minimize the impedance matching criteria, we should select $|\alpha |$ to be very small, but to maximize the matching network bandwidth, we should select $|\alpha |$ to be large. As for the angle, the impedance matching criterion tends to be minimized for negative values of $\angle \alpha$, while the matching network bandwidth is maximized for $\angle \alpha$ near 0. As a tradeoff between these two criteria, we select $|\alpha |=0.98$ and $\angle \alpha =-\pi /32$.

### Experimental results

**Fig. 3:**
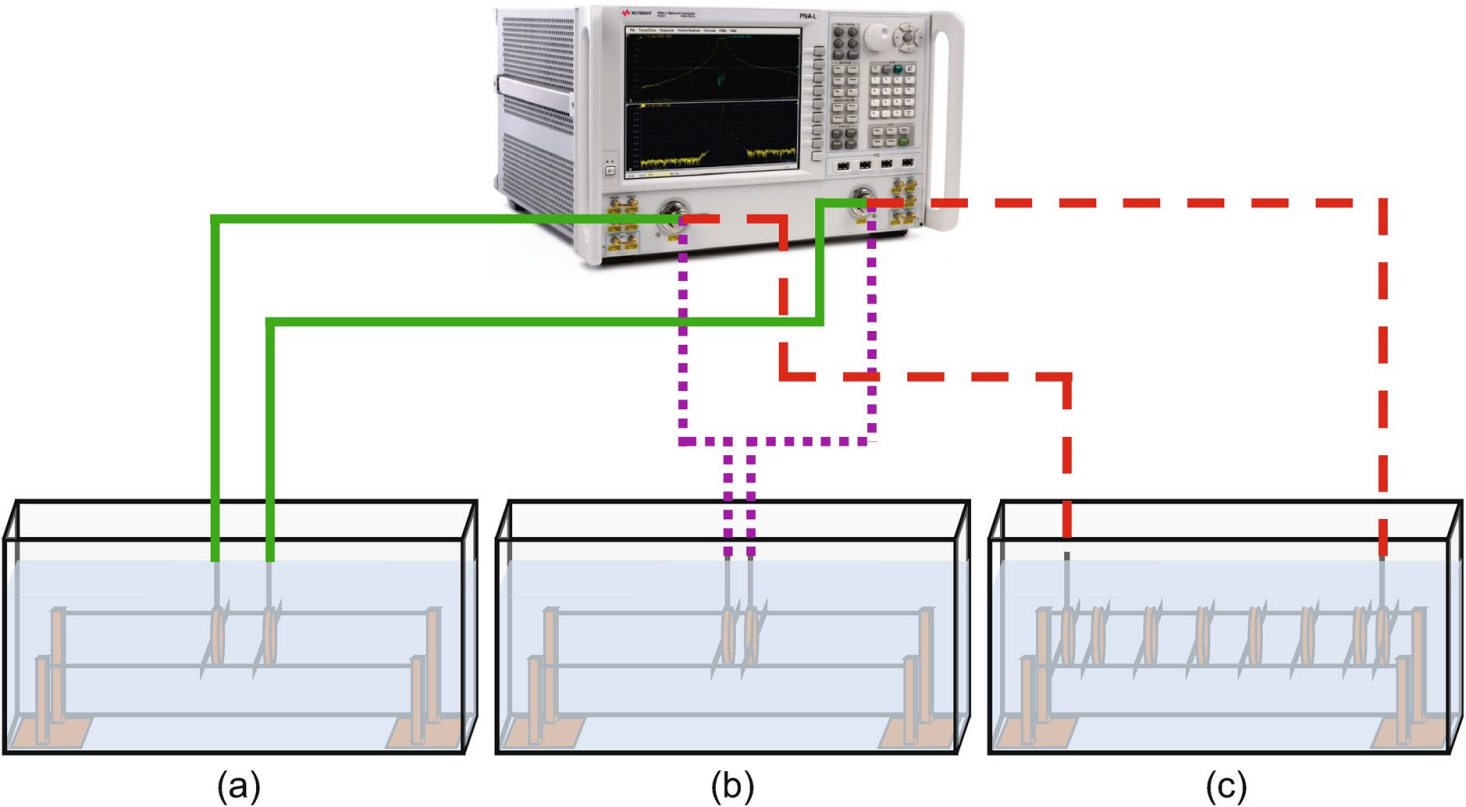
Diagram of experimental setups for S-Parameter measurements with a Keysight PNA-L N5235A. In each case, the stand-rod system with the corresponding device under test is submerged in a tub of salt water. Cables are routed from the PNA-L to the appropriate locations and connected to the device through water proof nozzles that extend outside the water. (**a**) Setup for extraction of complex-valued circuit parameters for element-element coupling at a distance of 2.25 cm. (**b**) Setup for extraction of complex-valued circuit parameters for element-transducer coupling at a distance of 1.82 cm. (**c**) Full system setup containing two transducers terminating the 6 element MIW. In this case, although not shown, the cables are connected to a matching network which, in turn, is connected to the transducer itself. The matching network is contained with the transducer and submerged.

To confirm the simulated results and examine the real-world performance of the underwater ocean communication system, we encapsulate the elements in 3D printed cases and utilize a stand-rod system to ensure alignment and uniform distances, similar to the system presented in literature^26^. The encapsulated elements and stand are then submerged in salt water and S-Parameter measurements are taken for a series of experiments. Figure 3 shows a diagram of each experiment.

First, the equivalent circuit parameters are extracted for the MIW system by utilizing two elements placed first at a distance of 2.25 cm and then at a distance of 1.82 cm apart representing the element-element coupling scenario and element-transducer coupling scenario, respectively, as shown in Fig. 3a and b. The circuit parameters are then extracted from the measured S-Parameters. At 40 MHz, we have $R=R_t=0.38$ $\Omega$, $L=L_t=136.4-j5$ nH, $M=6.07-j1.8$ nH and $M_t=7.90-j1.97$ nH which leads to $\kappa =0.09-j0.02$, $\tilde{Q}=90.9-j3.33$, and $\Lambda =\Lambda _t=-0.04$. The frequency-dependent data is discussed in Supplementary Note 3 with results shown in Supplementary Fig. 4. These data show good agreement with the simulated parameters, indicating that the distances and alignment are accurate, and that the encapsulation and stand-rod system have little effect on the results versus the simulated ideal encapsulation. With these measured results in mind, along with the availability of capacitors, each element and transducer are loaded with 112 pF capacitance by placing two 56 pF capacitors in parallel with one another. This helps achieve resonance at 40.7 MHz while reducing the additional series resistance introduced by the lumped elements. Finally, with the experimentally extracted circuit parameters and our previous selection of $|\alpha |=0.98$ and $\angle \alpha =-\pi /32$, we have $Z_L=2.45-j0.76$ as the optimal load impedance.

**Fig. 4:**
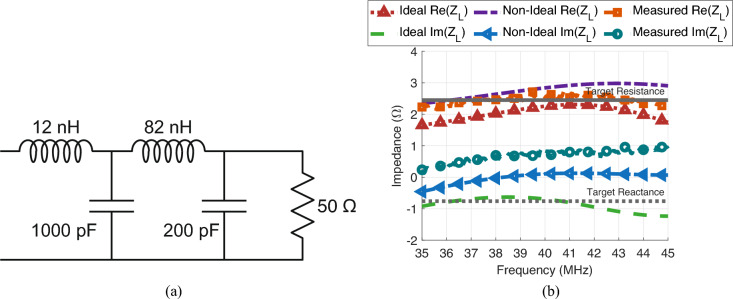
Matching network architecture and comparison of input impedance values. (**a**) Circuit diagram of matching network architecture with the 50 $\Omega$ load (right) representing the connection to a 50 $\Omega$ system and the open circuit (left) being the connection to the transducer itself. (**b**) Impedance versus frequency plot showing the calculated input impedance for (1) the ideal matching network, (2) the matching network with estimated non-idealities in the form of series resistors and expected component errors and (3) the manufactured and measured matching network. Here, the input impedance is defined at the transducer connection.

Next, a 2 section matching network is designed using lumped elements to transform the 50 $\Omega$ port impedance to the required $Z_L=2.45-j0.76$ $\Omega$. Each section is selected across our available components via brute-force optimization to achieve the desired $Z_L$ from 37.5 to 42.5 MHz. The final network architecture is shown in Fig. 4a. Here, we note that the 50 $\Omega$ load represents the port impedance, while the open circuit indicates the eventual connection point to the transducers and the reference point for determining $Z_L$. The network is then measured, and non-idealities, such as component series resistance and errors in value, are estimated. A comparison of the input impedance of the target, ideal, non-ideal, and measured circuits is shown in Fig. 4b. For the real component of the input impedance, the ideal, non-ideal, and measured circuits achieved 2.25, 2.84 and 2.58 $\Omega$ versus the target of 2.45 $\Omega$ at 40 MHz. As for the imaginary component of the input impedance, we have $-0.69$, 0.11 and 0.81 $\Omega$ for the ideal, non-ideal, and measured circuits, respectively, compared to the target of $-0.76$ $\Omega$. In general, the measured input impedance is close to the expected value from the circuit model, particularly when additional losses and component value errors are included and when the overall magnitude of the impedance is considered.

With the full system defined, the use of the proposed impedance matching model can be validated. To do so, a 6-element MIW with the previously defined geometry is created. This MIW is terminated on both ends with the transducers at the appropriate distance, as previously described. Both transducers are connected directly to individual matching networks, which are, in turn, connected to a vector network analyzer. The full experimental setup is diagrammed in Fig. 3c.

**Fig. 5:**
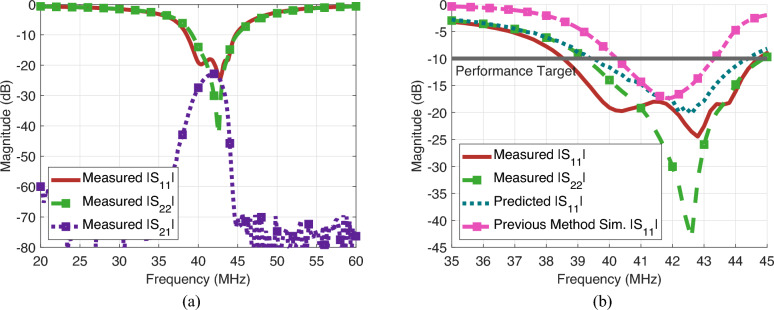
Impedance matched system performance based on proposed impedance matching model. (**a**) Magnitude of measured S-Parameter versus frequency from 20 to 60 MHz. Because the system is reciprocal, $|S_{12}|$ results are omitted. (**b**) Comparison of measured input/output reflection coefficients, predicted performance, and simulated performance following the previous state-of-the-art method^48^ versus frequency from 35 to 45 MHz. Additionally, the − 10 dB point is labeled as the target performance for acceptable impedance matching. The presented measured results are identical in both (**a**) and (**b**).

The S-Parameters are then measured and shown in Fig. 5. Note that the system is reciprocal andn as suchn the $|S_{12}|$ results are withheld. With the introduction of the transducer, the system achieves a minimum propagation loss of 1.53 dB/cm and a − 10 dB transmission bandwidth of 4.6 MHz between 39 and 43.6 MHz. This is an improvement of 0.69 dB/cm and 0.4 MHz when no matching network is present. As for the impedance matching, the $|S_{11}|$ minimum value is − 24.48 dB with a − 10 dB bandwidth of 6 MHz from 38.6 to 44.6 MHz. Because of manufacturing differences between matching networks, the $|S_{22}|$ is slightly different, with a minimum value of − 43.4 dB and a − 10 dB bandwidth of 5.4 MHz extending from 39.4 to 44.8 MHz. Both $|S_{11}|$ and $|S_{22}|$ are below − 9 dB for the entirety of the propagation passband, and have values of − 19.4 and − 30.0 dB at the frequency of minimum loss. These results indicate that the proposed model is highly effective as an impedance matching tool for MIWs in conductive media.

Figure 5b compares the $|S_{11}|$ and $|S_{22}|$ measurements with the predicted $|S_{11}|$ and the simulated $|S_{11}|$ following the previous state-of-the-art impedance matching method^48^. The simulated results are created by following the same design procedure presented here while replacing the proposed model with the current model available in literature^48^. The predicted model is created via a cascade of estimated S-Parameters based on the measured equivalent circuit parameters and the matching network with estimated non-idealities. The predicted model shows a − 10 dB bandwidth of 5 MHz, from 39.4 to 44.4 MHz, with a minimum value of − 20.2 dB. This predicted bandwidth is marginally smaller than the measured value, while the minimum value is slightly higher. These differences can primarily be attributed to non-ideal components on both the elements and the matching networks. Despite these differences, it is clear that the system performance can be accurately predicted based solely on the equivalent circuit parameters of the MIW and the matching network architecture. Comparing the measured system performance to the simulated results attained by using the previous state-of-the-art, it is clear that our method performs significantly better even when accounting for all non-idealities present in an experimental setting versus simulation. The previous method attains a − 10 dB bandwidth of only 3.1 MHz and a minimum value of − 17.3 dB which is a substantial decrease in both bandwidth and overall matching quality.

## Conclusion

In this work, we proposed and derived a new broadband impedance matching model for MIWs in conductive media. While the novel model utilized a similar derivation process as the lossless free-space case, the introduction of complex-valued parameters due to eddy current effects required the use of several approximations and led to the creation of three unique matching criteria. Each criterion was shown that it can be used independently from one another to determine the optimal transducer parameters with respect to a given MIW design. To validate the model and without loss of generality, we walked through the design process for a MIW communication system operating at 40 MHz in ocean water. First, the proposed matching criteria were optimized along with matching network considerations to determine the system parameters. Then, the experimental system was constructed, measured, and analyzed to ensure agreement with predicted results. Finally, the full system was constructed and tested. Our results showed a 15.5% fractional bandwidth, a maximum $|S_{11}|$ of − 9 dB within the propagation band, and a minimum propagation loss 1.53 dB/cm, with the impedance matching performance, showing excellent agreement with model predictions.

The proposed model significantly expands the ability to integrate MIWs into traditional RF systems. Previously, integration efforts were stifled by the inability to accurately handle losses, both in the MIW and in the environment. With the proposed model, there are no longer limitations with respect to (1) environmental factors and (2) MIW losses. In particular, this model enabled a novel demonstration of broadband communication of a lossy MIW in the traditionally challenging environment of ocean water. This technology can be used to create dense low-loss underwater sensor networks and enable multi-personnel short range wireless networks underwater, among other applications. Additionally, with the robust handling of eddy current effects, this model can also be applied to lower conductivity environments, such as underground or on the body.

While the impedance matching model is complete, there are system-level factors that still must be considered. Future work should focus on the mitigation of integration challenges, such as the small load impedance hurdle that was highlighted in this work. Similarly, the reverse design process can be demonstrated, i.e., designing an MIW to integrate into a given RF system and transducer. Finally, while we demonstrated the use of the impedance matching model alongside matching network considerations, a full co-design procedure for the RF system, MIW, and transducer can be created to further enhance system performance in challenging environments.

## Methods

### Simulation setup

All simulations are conducted in CST Studio using the frequency domain solver and adaptive tetrahedral mesh generation with a minimum of 10 passes. Because the elements are electrically small, the meshing is increased to a maximum tetrahedral size of 0.05 wavelengths on the elements. For all relevant simulations, the background material is set to relative permittivity, $\epsilon _r=74$ and conductivity, $\sigma =5$ S/m. The elements and transducers are encapsulated in thin volumes of air to eliminate conduction currents in the background material. Lumped elements are used to implement capacitors, while discrete ports are used to feed the system with a 1 W input signal. S-Parameter measurements are used to extract all relevant parameters.

### Experimental setup

First, the ocean water phantom is created by dissolving 820 g of salt in 6 gallons of distilled water at  25 °C^54^. This corresponds to approximately $\sigma =5$ S/m to mimic salt water^51^. Polylactic acid (PLA) based 3D printing resin is used to create a series of molds to (1) maintain the element shape and size, and (2) ensure waterproofing of the elements and transducers to eliminate conduction currents in the ocean water phantom. In addition, the thickness of the molds is minimized to maximize the amount of water between each element. We note that the molds used for the transducers are slightly thicker than the molds used for the elements, in order to accommodate both the matching network circuitry and the snorkel used to route the measurement cable underwater. Each mold is sealed using commercial hot-melt adhesive (HMA). The stand-rod system is created using the same PLA resin and the same HMA used to attach each encapsulated transducer and element to the system at the correct distance and alignment. Small metallic weights are placed on the bottom of the stand-rod system to keep the system from floating. The weights are sufficiently far away from the MIW and transducers to mitigate any effects that their presence would have on the results. Measurements are taken with a Keysight PNA-L N5235A via SMA connectors and cables. For more detail see Supplementary Note 2 and Supplementary Figs. 1–3. Repeatability of the experimental setup is discussed in Supplementary Note 4 with variability results shown in Supplementary Fig. 5.

### Predicted S-parameter model

The predicted S-Parameter model is formed in three subsystems. First, the full frequency-dependent extracted equivalent circuit parameters are used to determine the complex propagation constant of the MIW via Eq. (1) which, in turn, is inserted into Eq. (2). This characteristic impedance is then used to model the MIW as a lossy dispersive transmission line of length $(N-2)a$. Next, the terminal elements of the MIW are modeled separately in conjunction with the coupled transducers as an equivalent T-circuit. Finally, the matching network architecture is modeled along with estimated non-idealities in the form of series resistors with each component. Each subsystem is then placed in cascade in the appropriate order, and the overall system S-Parameters are calculated based on this cascade.

### Extraction of circuit parameters

Converting from S-Parameters to the complex-valued equivalent circuit parameters, $L_c=L_c'+jL_c''$ and $M_c=M_c'+jM_c''$, is done in two steps. First, the S-Parameters of a single unit in free-space are converted to Z-Parameters, ${\mathbf{Z}}$. From here, the free-space resistance, $R_0$ is taken as $Re(\mathbf {Z_{11}})$. Second, the S-Parameters of the two unit system in the environment of interest are converted to Z-Parameters and the parameters are calculated following the form in Eq. (19).

19 $$\begin{aligned} {\mathbf{Z}} = \begin{bmatrix} R_0 + j\omega (L_c'+jL_c'') j\omega (M_c'+jM_c'') \\ j\omega (M_c'+jM_c'') R_0 + j\omega (L_c'+jL_c'') \end{bmatrix} \end{aligned}$$

Note that in the above process we assume that the two units are identical. This is the case for both our element-element circuit parameters and our element-transducer circuit parameters. The process can be easily modified to accommodate two unique units.

## Electronic supplementary material

Below is the link to the electronic supplementary material.

Supplementary Information

## Data Availability

The data supporting the findings of this paper are available upon reasonable request to the corresponding author, C.J.
