# Supplementary material for: Broadband impedance matching for lossy magnetic metamaterials in conductive media
Source: Sci Rep. 2025 Jul 30;15:27778. doi: 10.1038/s41598-025-11452-6 (PMC12311141; doi:10.1038/s41598-025-11452-6)
Supplement: Supplementary file 1 — Supplementary Information [file 41598_2025_11452_MOESM1_ESM.pdf]

Broadband Impedance Matching for Lossy Magnetic Metamaterials in Conductive Media Supplementary Information

Connor Jenkins*[1] and Asimina Kiourti[1]

[1]ElectroScience Laboratory. Department of Electrical and Computer Engineering, The Ohio State University, Columbus OH, 43212, USA

Supplementary Note 1: Detailed Derivation of Impedance Matching Model

Circuit Model in Conductive Media

The impedance matching model relies on several assumptions: 1) nearest neighbor coupling only, 2) elements and transducers are electrically small, 3) each element is identical and uniformly spaced. With this in mind, we examine the case of a mono-atomic lossy MIW with geometric period a placed in a conductive non-magnetic medium with finite permeability. Each element is electrically isolated from the conductive media such that no conduction current is generated. Each element is defined by an impedance of $Z = R + j\omega L + 1/j\omega C$ where L is the self-inductance, C is the capacitance, R is the resistance of each element, ω is the radial frequency, and j is the imaginary unit. Finally, each element is coupled to its nearest neighbor by the mutual inductance M. To handle the eddy current effects of the surrounding conductive media, we represent the self- and mutual-inductances as complex-valued Kirchhoff coefficients[1]. Specifically, we define $L = L' + jL''$ and $M = M' + jM''$, where we note that, unlike the free space case, all values are weakly frequency-dependent. In this case, the dispersion relation for the MIW is[1]

$$1 - \frac{\omega_0^2}{\omega^2}(1 + j\Lambda)^{-1} - \frac{j}{\tilde{Q}} + \kappa \cos\gamma a = 0 \tag{1}$$

where γ is the complex propagation constant, $\tilde{Q} = \omega L/R$ is the complex-valued quality factor, $\kappa = 2M/L$ is the complex-valued coupling coefficient, $\Lambda = L''/L'$ is referred to as the cotangent factor, and $\omega_0 = 1/\sqrt{L'C}$ is the resonant angular frequency.

Next, we need to define the characteristic impedance of the MIW in question. To do so, we terminate the final element of an MIW with a load, Z_0. The circuit equation for this final element is

$$(j\omega L + 1/j\omega C + R + Z_0)\, I_n + j\omega M I_{n-1} = 0 \tag{2}$$

where the current in the nth element is defined as $I_n = Ie^{-j\gamma na}$. Just as with any transmission-line-like technology, the characteristic impedance of the line can be defined as the terminating impedance that eliminates reflections on the line. To enforce this behavior, we want the terminated circuit equation in Equation 2 to be identical to the circuit equation of the nth element in an infinite length line. As such we have

$$(j\omega L + 1/j\omega C + R + Z_0)\, I_n + j\omega M I_{n-1} = (j\omega L + 1/j\omega C + R)\, I_n + j\omega M I_{n-1} + j\omega M I_{n+1} \tag{3}$$

Using our current definition, this expands to

$$(j\omega L + 1/j\omega C + R + Z_0)\, Ie^{-j\gamma na} + j\omega M Ie^{-j\gamma(n-1)a} = (j\omega L + 1/j\omega C + R)\, Ie^{-j\gamma na} + j\omega M Ie^{-j\gamma(n-1)a} + j\omega M Ie^{-j\gamma(n+1)a} \tag{4}$$

Solving Z_0 then gives us the characteristic impedance of a lossy mono-atomic MIW in conductive media

$$Z_0 = j\omega M e^{-j\gamma a} \tag{5}$$

From here, the derivation follows similar steps to the free-space lossless case as defined in literature[2]. We examine a N element MIW defined above terminated with a transducer of self-impedance $Z_t = R_t + j\omega L_t + 1/j\omega C_t$ and loaded with an additional impedance $Z_L = R_L + jX_L$ that is coupled to the $0th$ element with mutual inductance $M_t = M_t' + jM_t''$. Note that the self-inductance of the transducer is also complex valued, with $L_t = L_t' + jL_t''$, such that the resonant frequency of the transducer is defined as $\omega_{0t} = 1/\sqrt{L_t'C}$. By defining the current in the nth element as I_n and the current in the transducer as I_L we have the following circuit equations.

$$(j\omega L + 1/j\omega C + R)\, I_0 + j\omega M I_{-1} + j\omega M_t I_L = 0 \tag{6}$$

$$(j\omega L_t + 1/j\omega C_t + R_t + Z_L)I_L + j\omega M_t I_0 = 0 \tag{7}$$

Solving for I_L in Equation 7 and substituting into Equation 6 yields

$$\left(j\omega L + 1/j\omega C + R + \frac{\omega^2 M_t^2}{Z_L + R_t + j\omega L_t(1 - \frac{\omega_{0t}^2}{\omega^2}(1+j\Lambda_t)^{-1})}\right) I_0 + j\omega M I_{-1} = 0 \tag{8}$$

where $\Lambda = L_t''/L_t'$. Using Equation 8, we define the effective load impedance as

$$Z_{L,eff} = \frac{\omega^2 M_t^2}{Z_L + R_t + j\omega L_t(1 - \frac{\omega_{0t}^2}{\omega^2}(1+j\Lambda_t)^{-1})} \tag{9}$$

Finally, we define the effective current reflection coefficient at the effective load

$$\Gamma_{eff} = -\frac{Z_{L,eff} - Z_0}{Z_{L,eff} + Z_0^*} \tag{10}$$

where * denotes the complex conjugate.

Impedance Normalization

To aid in the development of the impedance matching criteria, we normalize both Z_0 and $Z_{L,eff}$ by $\omega_0 M$ which is approximately the characteristic impedance of the MIW at the resonant frequency. For the characteristic impedance, we have

$$Z_{0N} = \frac{Z_0}{\omega_0 M} = \frac{w}{\sin(\gamma a) - j\cos(\gamma a)} \tag{11}$$

where $w = \omega/\omega_0$. Equation 1 is used to solve for both $\sin(\gamma a)$ and $\cos(\gamma a)$ which are substituted back into Equation 11 to get

$$Z_{0N} = \frac{w}{\sqrt{1 - (-\frac{1}{\kappa} + \frac{1}{\kappa w^2(1+j\Lambda)} + \frac{j}{\kappa\tilde{Q}})^2} + j(\frac{1}{\kappa} - \frac{1}{\kappa w^2(1+j\Lambda)} - \frac{j}{\kappa\tilde{Q}})} \tag{12}$$

Likewise, for the load impedance, we have

$$Z_{LN,eff} = \frac{Z_{L,eff}}{\omega_0 M} = \frac{\omega^2 M_t^2}{\omega_0 M(Z_L + R_t + j\omega L_t(1 - \frac{\omega_{0t}^2}{\omega^2}(1+j\Lambda_t)^{-1}))} \tag{13}$$

This is then simplified using the following normalized parameter definitions $\mu = M_t/M$, $\lambda = L_t/L$, $\eta = \omega_{0t}/\omega_0$, and $\rho_T = (R_t + Z_L)/\omega_0 M$ to yield

$$Z_{LN,eff} = \frac{w}{\frac{\rho_T}{w\mu^2} + j\frac{2\lambda}{\kappa\mu^2}(1 - \frac{\eta^2}{w^2}(1+j\Lambda_t)^{-1})} \tag{14}$$

Binomial Approximation

To eliminate reflections, we want $Z_{L,eff} - Z_0 = 0$ which is equivalent to $Z_{LN,eff} - Z_{0N} = 0$ and $Y_{LN,eff} - Y_{0N} = 0$ where $Y_{LN,eff} = 1/Z_{LN,eff}$ and $Y_{0N} = 1/Z_{0N}$. Utilizing the normalized admittances, we have

$$\sqrt{1 - \left(-\frac{1}{\kappa} + \frac{1}{\kappa w^2(1+j\Lambda)} + \frac{j}{\kappa\tilde{Q}}\right)^2} + j\left(\frac{1}{\kappa} - \frac{1}{\kappa w^2(1+j\Lambda)} - \frac{j}{\kappa\tilde{Q}}\right) - \frac{\rho_T}{w\mu^2} - j\frac{2\lambda}{\kappa\mu^2}\left(1 - \frac{\eta^2}{w^2}(1+j\Lambda_t)^{-1}\right) = 0 \tag{15}$$

which is our impedance matching expression. While this is sufficient to determine if a given design achieves strong impedance matching, further simplifications will lead to several equations that enlighten the design process itself. To create the impedance matching criteria, we will separate the terms that include the 90°phase shift introduced by $\pm j$ from those terms that do not have an additional phase change. We will refer to these components as the quadrature and in-phase components, respectively. To

separate these components, we utilize the binomial approximation on the terms under the radical. Our conditions to use this approximation are

$$\left|\left(-\frac{1}{\kappa}+\frac{1}{\kappa w^2(1+j\Lambda)}+\frac{j}{\kappa\tilde{Q}}\right)^2\right|<1 \tag{16}$$

$$\frac{1}{2}\left|\left(-\frac{1}{\kappa}+\frac{1}{\kappa w^2(1+j\Lambda)}+\frac{j}{\kappa\tilde{Q}}\right)^2\right|\ll 1 \tag{17}$$

Using this approximation, we have

$$1-\frac{1}{2}\left(-\frac{1}{\kappa}+\frac{1}{\kappa w^2(1+j\Lambda)}+\frac{j}{\kappa\tilde{Q}}\right)^2+j\left(\frac{1}{\kappa}-\frac{1}{\kappa w^2(1+j\Lambda)}-\frac{j}{\kappa\tilde{Q}}\right)-\frac{\rho_T}{w\mu^2}-j\frac{2\lambda}{\kappa\mu^2}\left(1-\frac{\eta^2}{w^2}(1+j\Lambda_t)^{-1}\right)=0 \tag{18}$$

Quadrature and In-Phase Components

We expand the expression and group our terms by quadrature and in-phase components. Doing so, gives us

$$\begin{aligned}
&1-\frac{1}{2\kappa^2}+\frac{1}{2\kappa^2\tilde{Q}^2}-\frac{\Lambda}{\kappa^2\tilde{Q}w^2(\Lambda^2+1)}+\frac{\Lambda^2}{2\kappa^2w^4(\Lambda^4+2\Lambda^2+1)}-\frac{1}{2\kappa^2w^4(\Lambda^4+2\Lambda^2+1)}\\
&+\frac{1}{\kappa^2w^2(\Lambda^2+1)}-\frac{\Lambda}{\kappa w^2(\Lambda^2+1)}+\frac{1}{\kappa\tilde{Q}}-\frac{\rho_T}{w\mu^2}+\frac{2\lambda\eta^2\Lambda_t}{\kappa\mu^2w^2(\Lambda_t^2+1)}\\
&+j\left(\frac{1}{\kappa^2\tilde{Q}}-\frac{1}{\kappa^2\tilde{Q}w^2(\Lambda^2+1)}+\frac{\Lambda}{\kappa^2w^4(\Lambda^4+2\Lambda^2+1)}-\frac{\Lambda}{\kappa^2w^2(\Lambda^2+1)}\right)\\
&+j\left(\frac{1}{\kappa}-\frac{1}{\kappa w^2(\Lambda^2+1)}-\frac{2\lambda}{\kappa\mu^2}+\frac{2\lambda\eta^2}{\kappa\mu^2w^2(\Lambda_t^2+1)}\right)=0
\end{aligned} \tag{19}$$

Focusing on the quadrature components, we can simplify the expression and group by frequency component

$$\begin{aligned}
&w^4\left(\frac{1}{\tilde{Q}}(\Lambda^2+1)(\Lambda_t^2+1)(\Lambda^4+2\Lambda^2+1)+\kappa(\Lambda^2+1)(\Lambda_t^2+1)(\Lambda^4+2\Lambda^2+1)-\frac{2\lambda\kappa}{\mu^2}(\Lambda^2+1)(\Lambda_t^2+1)(\Lambda^4+2\Lambda^2+1)\right)+\\
&w^2\left(\frac{2\lambda\eta^2\kappa}{\mu^2}(\Lambda^2+1)(\Lambda^4+2\Lambda^2+1)-\frac{1}{\tilde{Q}}(\Lambda_t^2+1)(\Lambda^4+2\Lambda^2+1)-\Lambda(\Lambda_t^2+1)(\Lambda^4+2\Lambda^2+1)-\kappa(\Lambda_t^2+1)(\Lambda^4+2\Lambda^2+1)\right)\\
&+\Lambda(\Lambda^2+1)(\Lambda_t^2+1)=0
\end{aligned} \tag{20}$$

To satisfy Equation 20 for all frequencies, we need each component to be equal to 0 as shown in Equations 21-23.

$$\begin{aligned}
&\frac{1}{\tilde{Q}}(\Lambda^2+1)(\Lambda_t^2+1)(\Lambda^4+2\Lambda^2+1)+\kappa(\Lambda^2+1)(\Lambda_t^2+1)(\Lambda^4+2\Lambda^2+1)-\\
&\frac{2\lambda\kappa}{\mu^2}(\Lambda^2+1)(\Lambda_t^2+1)(\Lambda^4+2\Lambda^2+1)=0
\end{aligned} \tag{21}$$

$$\begin{aligned}
&\frac{2\lambda\eta^2\kappa}{\mu^2}(\Lambda^2+1)(\Lambda^4+2\Lambda^2+1)-\frac{1}{\tilde{Q}}(\Lambda_t^2+1)(\Lambda^4+2\Lambda^2+1)-\\
&\Lambda(\Lambda_t^2+1)(\Lambda^4+2\Lambda^2+1)-\kappa(\Lambda_t^2+1)(\Lambda^4+2\Lambda^2+1)=0
\end{aligned} \tag{22}$$

$$\Lambda(\Lambda^2+1)(\Lambda_t^2+1)=0 \tag{23}$$

First, by setting the w^4 component in Equation 21 equal to 0, we have

$$\frac{\mu^2}{2}\left(\frac{1}{\kappa\tilde{Q}}+1\right)=\lambda \tag{24}$$

which relates the geometry of the MIW to the geometry of the transducer. Next, utilizing Equation 24 and solving Equation 22 gives us

$$\eta^2 = \frac{(1+\Lambda_t^2)(1/\tilde{Q}+\Lambda+\kappa)}{(1+\Lambda^2)(1/\tilde{Q}+\kappa)} \tag{25}$$

relating the geometry of the MIW to the resonant frequency of the transducer. While we now have three frequency-independent criteria in Equations 23-25, Equation 23 cannot be true in a conductive environment as it relies directly on Λ. Instead, we absorb the frequency-independent component in Equation 23 into the w^2 component in Equation 22 such that our new expression is

$$w^2\left(\frac{2\lambda\eta^2\kappa}{\mu^2}(\Lambda^2+1)(\Lambda^4+2\Lambda^2+1)-\frac{1}{\tilde{Q}}(\Lambda_t^2+1)(\Lambda^4+2\Lambda^2+1)-\Lambda(\Lambda_t^2+1)(\Lambda^4+2\Lambda^2+1)-\kappa(\Lambda_t^2+1)(\Lambda^4+2\Lambda^2+1)\right)+ \Lambda(\Lambda^2+1)(\Lambda_t^2+1)=0 \tag{26}$$

Applying the approximation, $\Lambda^2 \approx \Lambda_t^2 \approx 0$, to Equation 26 we have

$$\frac{2\lambda\eta^2\kappa}{\mu^2} = \left(1-\frac{1}{w^2}\right)\Lambda+\kappa+\frac{1}{\tilde{Q}} \tag{27}$$

Simplifying the expression and applying our previous criteria in Equation 24 gives us

$$\eta^2 = \frac{(1/\tilde{Q}+\kappa+\Lambda(1-1/w^2))}{(1/\tilde{Q}+\kappa)} \tag{28}$$

For near resonance conditions, where mono-atomic MIWs operate, we have $\Lambda(1-1/w^2)) \approx 0$. This is an even stronger approximation for $|\Lambda| < 1$ which is often the case. As such, our final criterion for the quadrature component of the impedance matching equation is

$$\eta^2 \approx 1 \tag{29}$$

Turning to the in-phase components, we have

$$\begin{aligned}1-\frac{1}{2\kappa^2}+\frac{1}{2\kappa^2\tilde{Q}^2}-\frac{\Lambda}{\kappa^2\tilde{Q}w^2(\Lambda^2+1)}+\frac{\Lambda^2}{2\kappa^2w^4(\Lambda^4+2\Lambda^2+1)}-\frac{1}{2\kappa^2w^4(\Lambda^4+2\Lambda^2+1)}\\+\frac{1}{\kappa^2w^2(\Lambda^2+1)}-\frac{\Lambda}{\kappa w^2(\Lambda^2+1)}+\frac{1}{\kappa\tilde{Q}}-\frac{\rho_T}{w\mu^2}+\frac{2\lambda\eta^2\Lambda_t}{\kappa\mu^2w^2(\Lambda_t^2+1)}=0\end{aligned} \tag{30}$$

By following the same procedure as the quadrature components, we can expand Equation 30 and group by frequency component, giving us

$$\begin{aligned}&w^4(1+\Lambda^2)^2(2\kappa^2+1/\tilde{Q}^2+2\kappa/\tilde{Q}-1)+w^3(-2\alpha\kappa^2(1+\Lambda^2)^2)+\\&2w^2(1+\Lambda^2)(1+\Lambda\Lambda_t-\Lambda_t/\tilde{Q}+\Lambda/\tilde{Q}-\kappa\Lambda+\kappa\Lambda_t)+(\Lambda^2-1)=0\end{aligned} \tag{31}$$

where $\alpha = \rho_T/\mu^2$. For a real MIW design, particularly in conductive media, Equation 31 cannot be satisfied for all frequencies, even if dramatic assumptions are made regarding the design. This aligns with the free-space lossless case that saw a similar criterion emerge[2]. As such, this equation will remain as is.

The final impedance matching criteria are shown in Eqns. 24, 29, and 31 based on approximations from the full expression shown in Equation 15.

Supplementary Note 2: Experimental Setup

This section is dedicated to highlighting the exact experimental setup used for all measured data. The 3D printed cases for the transducers and elements are shown in Fig. 1. Both cases have an inset groove on the center circle to shape the element and transducer to the appropriate shape and size. Adhesive tape is used to further secure the copper loops to the cases. The

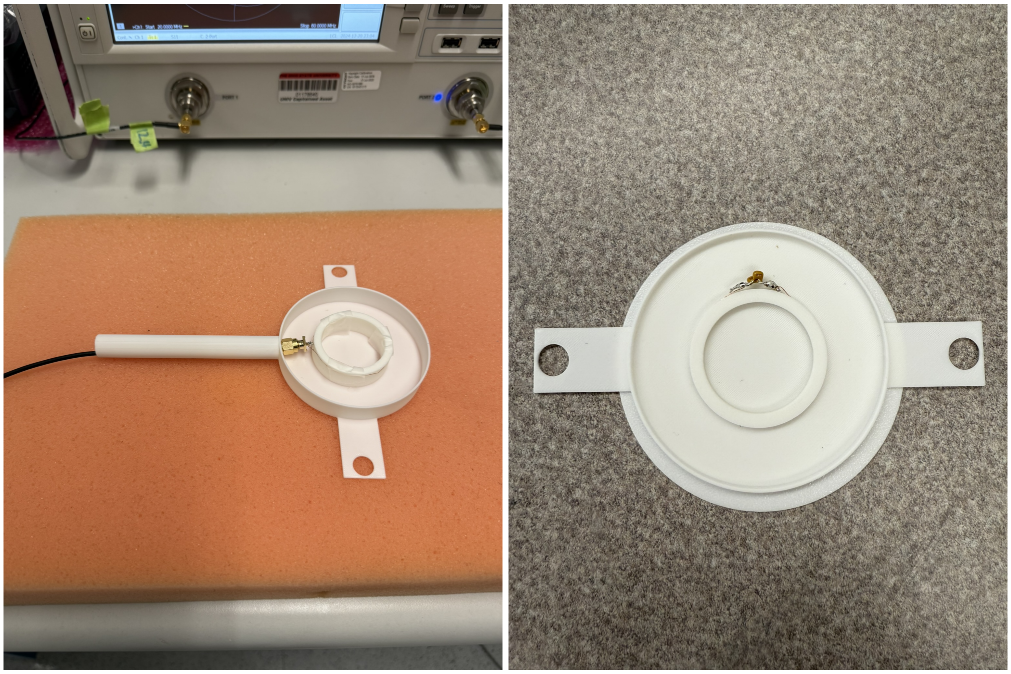

(a) (b)

Supplementary Figure 1. 3D printed cases used to encapsulate the transducer and elements for the underwater experiments: **(a)** transducer case showing the connection to the copper loop via SMA cable and connector, **(b)** element case showing the loop wrapped around the inset groove. Includes lumped element capacitor setup.

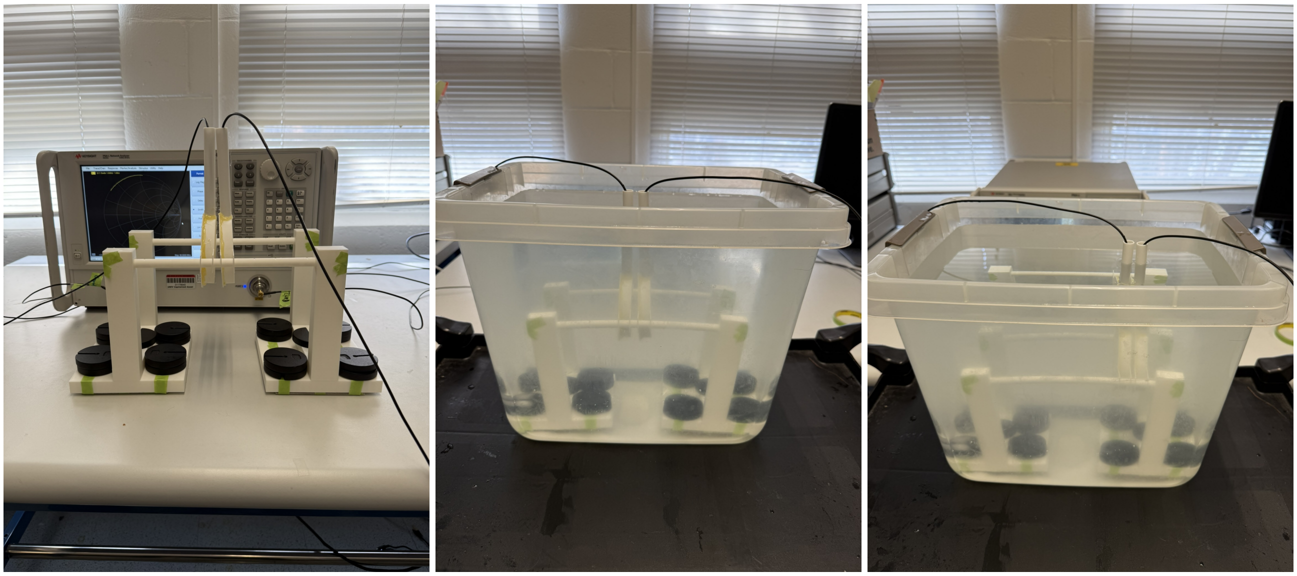

(a) (b) (c)

Supplementary Figure 2. Experimental setups for the extraction of measured equivalent circuit parameters. The stand-rod system and weights are used to align 3D printed cases and ensure accurate distances between devices. **(a)** Two elements spaced 2.25 cm apart measured in air, used solely for the extraction of the free-space resistance of elements and transducer. **(b)** Two elements spaced 2.25 cm apart measured in ocean water phantom, used to extract complex-valued self impedance and mutual inductance values for MIW design, **(c)** Two elements spaced 1.83 cm apart measured in ocean water phantom, used to extract the complex-valued mutual inductance between the final MIW element and the transducer.

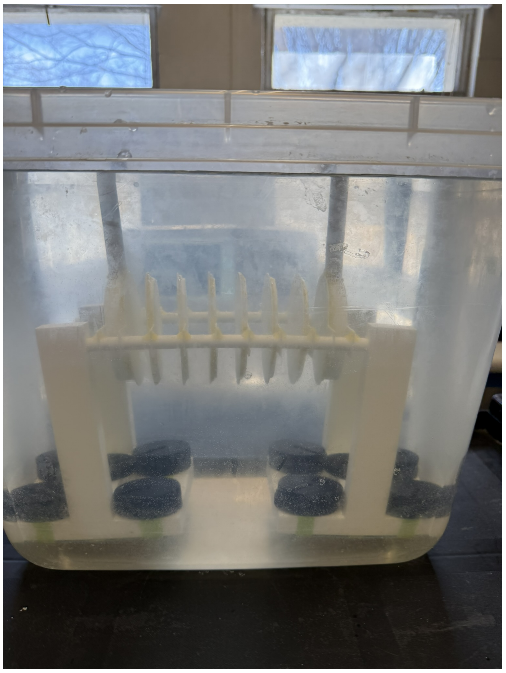

(a)

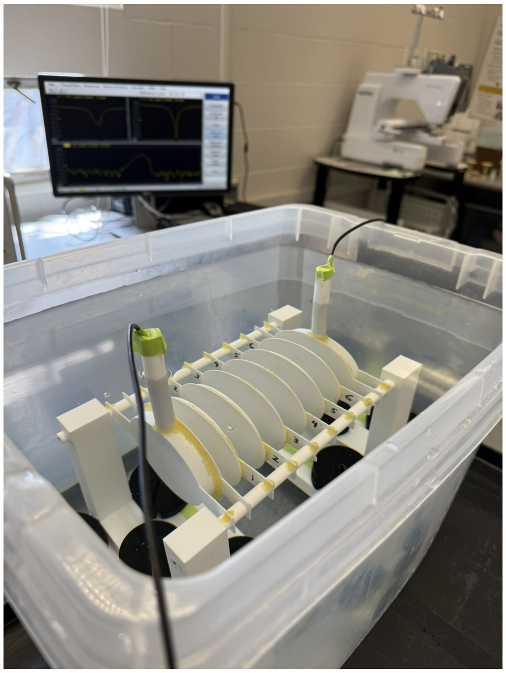

(b)

Supplementary Figure 3. Matched MIW system submerged in ocean water phantom. A 3D printed stand-rod system is used to align the transducers and elements while small weights are used to ensure that the system does not float. **(a)** Front view of matched MIW system showing water fully surrounding each element and transducer, **(b)** side view of matched MIW system highlighting the wires leaving the ocean water phantom via 3D printed snorkels and the distance between the system and the outer container walls.

transducer case is thicker than the element case to allow space for the SMA connector and matching network and includes a snorkel to allow the cable to safely exit the ocean water phantom.

The experimental setups used to extract the equivalent circuit parameters of the MIW are shown in Fig. 2. The 3D printed stand-rod system includes notches to accurately maintain the appropriate distance between loops. The in-air setup in Fig. 2a is used to extract the free-space resistance of the elements, while the ocean water phantom setups shown in Figs. 2b and 2c are used to extract the complex self-impedance of each element along with the two required mutual inductance values - element-element coupling and element-transducer coupling.

The experimental setup for the measurement of the matched MIW system is shown in Fig. 3. The system was designed such that the ocean water phantom fully surrounded each element and each transducer, while maximizing the space between the MIW, the outer container wall, and the required metal weights that ensure that the system did not float. The inclusion of the weights has little impact on the performance of the system as they are non-resonant and placed far enough away from the MIW. As such, the field strength is very small, especially when considering the attenuating properties of the ocean water phantom.

Supplementary Note 3: Frequency-Dependent Circuit Parameters

Using the experimental setups shown in Fig. 2, the complex valued equivalent circuit parameters are extracted from the S-parameter measurements and compared to simulated results. The comparison of the self-inductance, $L = L' + jL''$, and mutual inductance values, $M = M' + jM''$ and $M_t = M_t' + jM_t''$, across frequency are shown in Fig. 4, where L is the self-inductance of both the elements and transducers, M is the mutual inductance between two MIW elements, and M_t is the mutual inductance between the final MIW element and a transducer. Here, we see the weak frequency dependency of the parameters based on the eddy current effects of the surrounding ocean water phantom, particularly in the imaginary component of each parameter. In general, the measured imaginary components of the parameters show excellent agreement with the simulated results. The measured real components of the self-inductance are marginally larger than the simulated value, while the measured real components of both mutual inductance values are slightly lower than expected. The additional self-inductance is likely caused by the need to use slightly larger loops than ideal in order to connect the loops to connectors, while the slight decrease in mutual inductance can be attributed to slight misalignment of the elements.

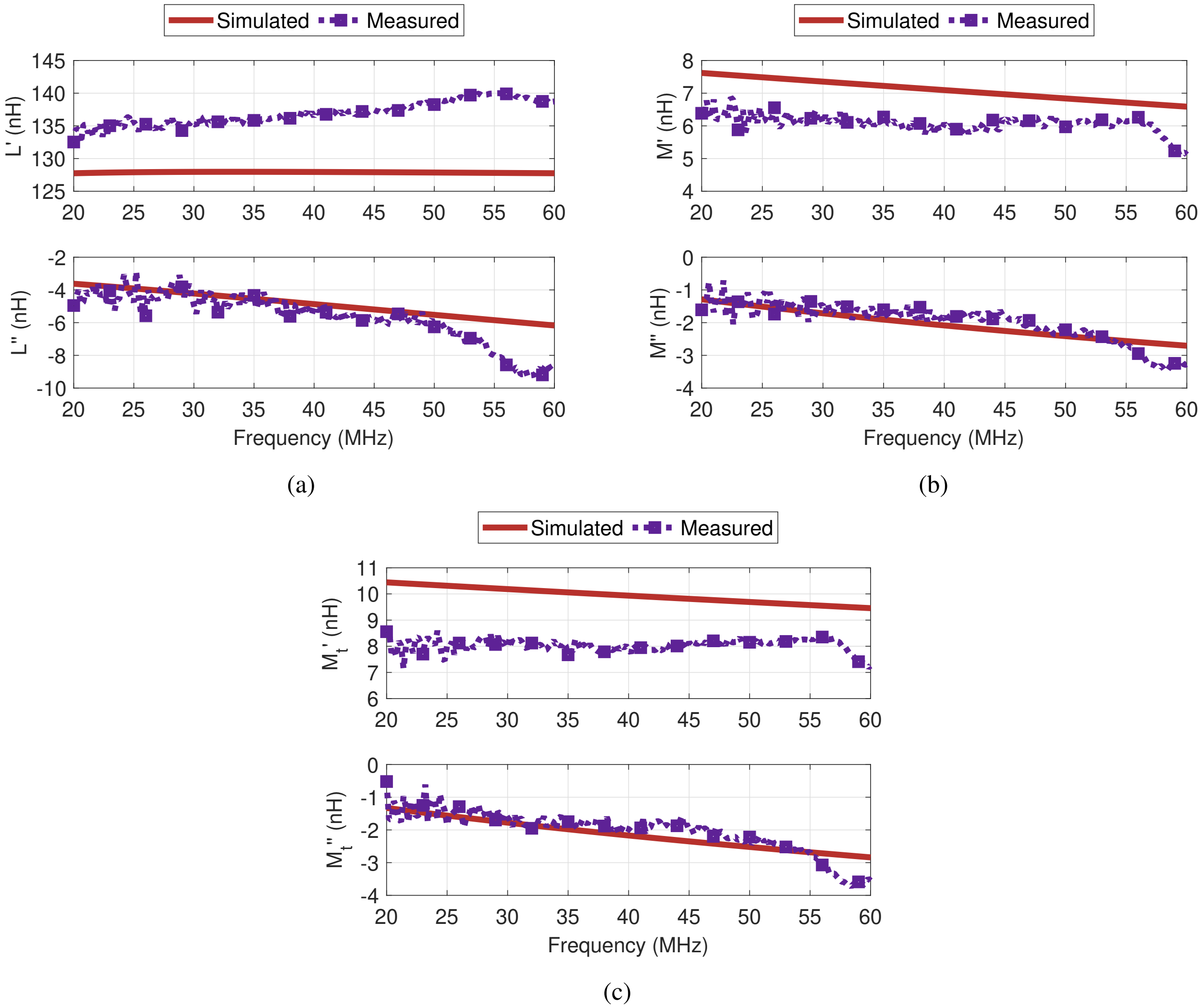

Supplementary Figure 4. Simulated versus measured equivalent complex-valued circuit parameters from 20 to 60 MHz. **(a)** Comparison of measured and simulated self-inductance, $L = L' + jL''$, of elements with a radius of 2 cm. Because the transducer geometry is identical to the elements, this is also the self-inductance of the transducers. **(b)** Measured and simulated mutual inductance, $M = M' + jM''$, between two MIW elements with a radius of 2 cm at a separation of 2.25 cm. **(c)** Mutual inductance in simulation and measurement between the final MIW element and the transducer, both loops of radius 2 cm, at a distance of 1.83 cm.

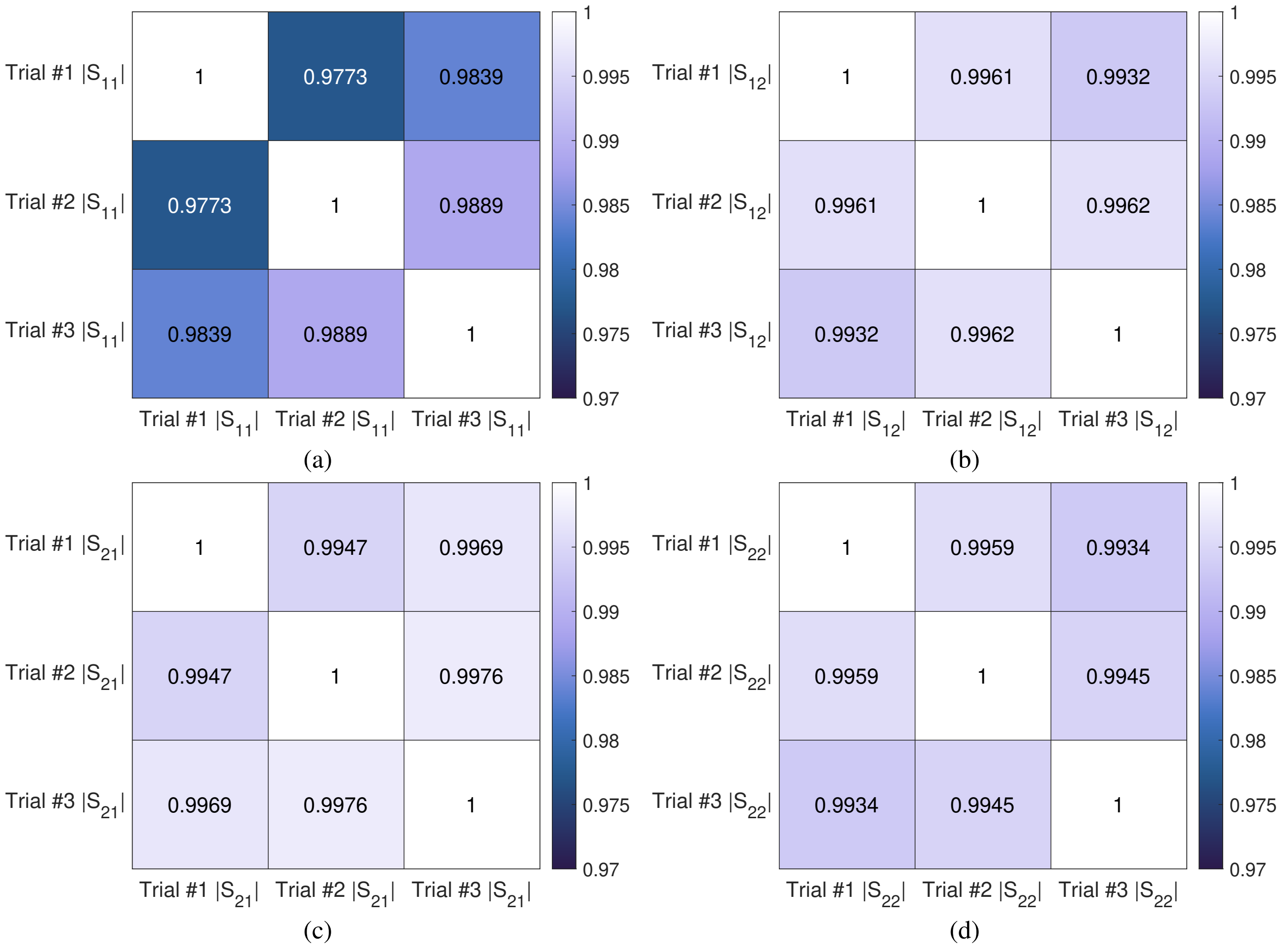

Supplementary Figure 5. Correlation matrices of the magnitude of S-parameters between 3 trials of randomly varying the MIW element order while keeping transducers constant. **(a)** Correlation matrix of $|S_{11}|$ showing a minimum correlation of 0.977 and a maximum correlation of 0.989. **(b)** Correlation of $|S_{12}|$ between trials with a minimum and maximum correlation of 0.993 and 0.996. **(c)** Correlation of $|S_{21}|$ with a minimum correlation of 0.995 and a maximum correlation of 0.998 **(d)** Correlation matrix of $|S_{22}|$ with a minimum correlation of 0.993 and a maximum correlation of 0.996.

Supplementary Note 4: Experimental Variability Study

To ensure that the outcomes of the matched MIW experiment is not heavily impacted by the handcrafted system, a small study is conducted. The primary focus of this study is on the ability to accurately place and align MIW elements by hand using the stand-rod system. To study this, the MIW system is tested without the inclusion of the matching network with an original element order in the ocean water phantom. Then, while the transducers remained constant, the order of the MIW elements is randomized and the system is re-measured. This process is repeated one more time, for a total of 3 trials. The experimental setup is similar to the scenario displayed in Fig. 3. Each trial required a complete removal of each element and one transducer from the stand rod system in order to then re-attach the elements in the new order. The correlation matrices of the magnitude of the measured S-parameters is shown in Fig. 5.

Across all four trials, the minimum correlation between trials is 0.977 while the maximum correlation between trials is 0.998. In general, the trials are highly correlated with one another, indicating that the stand-rod system works very effectively and maintains accurate positioning and alignment of MIW elements relatively to one another and to the transducers.

References

1. Chu, S. *et al.* Magnetoinductive waves in attenuating media. *Sci. Reports* **11**, 7679, DOI: 10.1038/s41598-021-85838-7 (2021).

2. Syms, R. R. A., Solymar, L. & Young, I. R. Broadband coupling transducers for magneto-inductive cables. *J. Phys. D: Appl. Phys.* **43**, 285003, DOI: 10.1088/0022-3727/43/28/285003 (2010).
